# Prognostic scoring systems-validation and their utility in patients with abdominal sepsis in colon peritonitis

**Published:** 2014-03-25

**Authors:** G Teleanu, F Iordache, M Beuran

**Affiliations:** "Floreasca" Emergency Clinic Hospital, Surgery Clinic

**Keywords:** prognostic scoring systems, abdominal sepsis, colon peritonitis, CR-POSSUM score

## Abstract

Aim: The purpose of this article is to analyze and validate the CR-POSSUM score in patients with peritonitis of colonic origin, considering mortality forecasting ability.

Methods: We conducted a prospective longitudinal study of the Surgery Clinic in the Emergency Hospital in Bucharest in January 2008-December 2011. Patients operated on for peritonitis of colonic origin were included in this study. The prognostic CR-POSSUM scores and Mannheim peritonitis index were calculated by using data from observation sheets. There have been a number of deaths and overall mortality was calculated.

Results: There were 58 patients with abdominal sepsis, hospitalized and operated, registering a 17.24% mortality rate. Time from admission until the time of operation was divided between up to 24 hours and over 24 hours, recording 27 (46.55%) cases operated <24 hours and 31 (53, 45%) of patients operated> for 24 hours. Operative severity scores were calculated by taking into account data provided by each surgeon on intraoperative appearance.

Conclusions: The results of this study demonstrated that both CR-POSSUM score and Mannheim have a prognostic value for patients with abdominal sepsis in colonic peritonitis, both being surgery scores.

## Introduction

The ideal score for complete surgical evaluation should assess both morbidity and mortality. It should also be quick and easy to use and apply to all types of surgery, both emergency and elective. Also the scoring system must be easily implemented in the existing assessment programs. 

 The literature describes numerous scoring systems that predict the mortality risk, but does not analyze postoperative morbidity. Some scores have been developed and are useful for certain types of surgery, others are used to assess the mortality risk and a little morbidity in certain groups of patients and the scoring system assesses the risk of developing complications. 

 When comparing the quality of care, morbidity and mortality rates have obvious limitations and false negative results may occur because the physiological state of the patient at the time of surgery, the complexity of the surgery, the patient's health status, age, etc., are elements which are not taken into account. 

 The scoring system is described; which has reduced the number of variables in order to facilitate scoring system, but this reduction certainly has some disadvantages. 

 Starting from this premise, Copeland et al. [**1**] developed a scoring system using 12 variables and showed that any reduction in the number of variables below, will affect the predictability of mortality and morbidity. This scoring system makes a prediction of morbidity and mortality for patients in general may be differences between groups of patients according to a certain type of surgery. 

 POSSUM (Physiological and Operative Severity Score for the Enumeration of Mortality and Morbidity) described in 1991 by Copeland is a widely used score. It uses 12 variables and 6 preoperative physiologic variables operators on a scoring system of 4 degrees, the results being analyzed by using a linear or exponential method. Exponential analysis method has a higher degree of accuracy, but is more difficult to achieve. 

 Since its first description, several changes have occurred, aiming to more accurately predicting morbidity and mortality. It can also be used for risk assessment in similar patient subgroups from different backgrounds, so that the result can be compared. 

 POSSUM score is the only score to be used purely for surgery. 

 Later described P-POSSUM score (Portsmouth POSSUM modification), considered superior in predicting mortality risk. P-POSSUM uses the same variable; the results being obtained by linear analysis. 

 CR-POSSUM score was described in 2004 and is intended solely for colorectal surgery.

## Method

We conducted a prospective longitudinal study of the Surgery Clinic in the Emergency Hospital in Bucharest, in January 2008-December 2011, by performing a retrospective analysis. 

Patients operated on for peritonitis of colonic origin were included in this study. There were 58 patients with abdominal sepsis, hospitalized and operated, registering a 17.24% mortality rate. Prognostic CR-POSSUM scores and Mannheim peritonitis index were calculated by using data from the observation sheets. Operative severity scores were calculated by taking into account the data provided by each surgeon on intraoperative appearance. There have been a number of deaths and overall mortality was calculated. 

 Using data obtained on expected mortality and morbidity, we calculated the ratio O / E (O / E ratio - the ratio of observed and expected cases). A report O / E of less than 1 means a supraprediction and report O / E greater than 1 is a subprediction. 

 Parameters for calculating CR-POSSUM scores were obtained in Table 1.

**Table 1:**
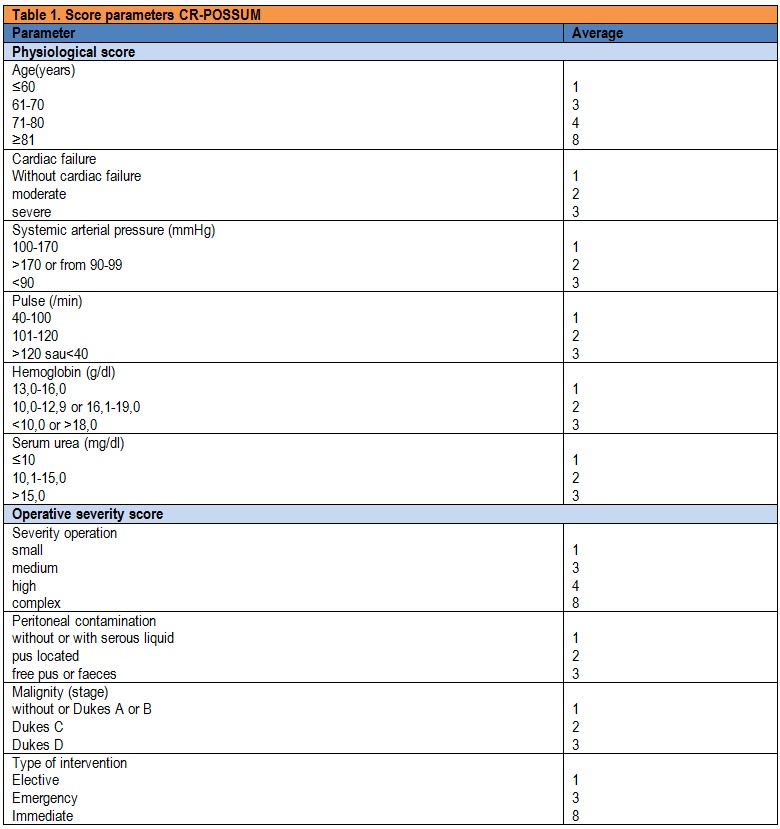
Score parameters CR-POSSUM

 Variables used to calculate the score Mannheim are presented in Table 2 and were obtained by using data from the observation charts using data provided by the surgeon, including the intraoperative exploration.

**Table 2:**
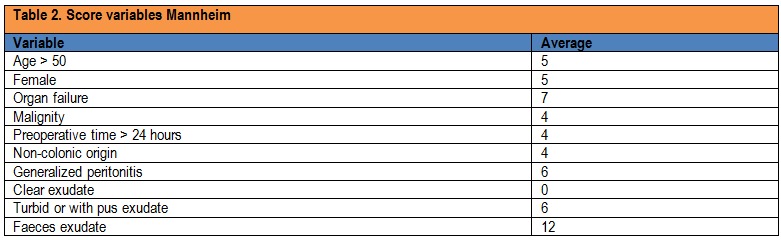
Score variables Mannheim

## Results

From January 2008 to December 2011 in the Surgery Clinic Emergency Hospital Bucharest 58 patients diagnosed with peritonitis of colonic origin were recorded. Patient age ranged between 44 and 84 years, with a mean of 66.68 ± 10.95 years. Distribution of cases according to the age group for the study group, showed an increased incidence for the decade 71-80 years (19 cases), followed by the decade 51-60 years (15 cases). Were recorded and 3 cases aged 41-50 years.

 There were 33 (56.89%) men and 25 (43.1%) women in the study. Note in the case of this group, like lots of previous studies, a higher proportion of male patients in the patients with colonic peritonitis.

**Fig. 1:**
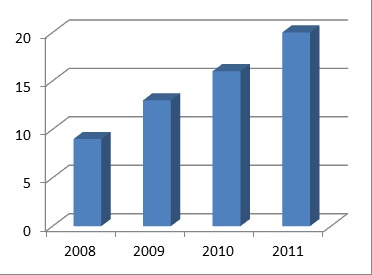
Distribution of cases

 Distribution of cases per year was 9 cases followed in 2008, 13 cases in 2009, 26 cases in 2010 and 20 cases in 2011. This distribution exemplified in Fig. 1 does not necessarily support an increased incidence of colonic peritonitis, but the fact that the parameters collection, some cases were excluded due to insufficient data obtained from the observation charts (in total there were 5 such cases, all first period studied).

Time from admission until the time of operation was divided between up to 24 hours and over 24 hours, recording 27 (46.55%) cases operated <24 hours and 31 (53, 45%) of patients operated > 24 hours. Depending on the type of surgery there were 11 (18.96%) immediate surgeries (under 2 hours), 16 (27.58%) emergency interventions (up to 24 hours) and 31 (53.45%) interventions considered elective. The presence of malignancy was observed in 36 patients (62%), and organ failure was present in 20 patients (34.48%). The extreme values of Hb ranged from 5.5 g / dl and 14.3 g / dL and serum urea had values between 35mg/dl and 212mg/dl. Of all the patients, there were 10 cases of localized peritonitis (17.24%) and 48 cases of generalized peritonitis (82.75%).

The most frequent complication was parietal suppuration in 24 cases (41.37%), followed by organ failure in 20 cases (34.48%). There were 13 (22.41%) cases of heart failure, seven (12%) cases of pulmonary complications, 4 (6.89%) patients underwent the surgical wound dehiscence that required reinterventions in 3 cases, 3 (5 17%) of dehiscence of the anastomosis, the reintervention in all patients.

 In the group of patients studied, calculate the CR-POSSUM physiological values showed minimum 6 and maximum of 18, with a mean of 11.18 ± 3.09. For operative severity score CR-POSSUM, the values were between 7 and 17 with a mean of 11.55 ± 2.94. Mannheim score values were calculated between 11 and 41, with a mean of 27.89 ± 6.1.

**Table 3:**
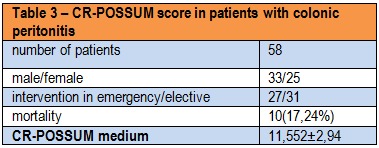
CR-POSSUM score in patients with colonic peritonitis

 There were 26 patients with a predicted mortality of less than 10% by 7 patients had a predicted mortality 10-20% and 40-50% and 50-100% forecast and 8 patients were associated.

 In the group of patients, mortality according to the age group ranged from 0 deaths for the decade 41-50 years and 4 deaths in patients over 81 years. Mortality predicted by CR-POSSUM score was 0 deaths for the 41-50 years group, corresponding decade peaked 71-80 years and reached 4 deaths attributed to patients in the group projected over 81 years. Report O:E is 1 in the decade 41-50 years, 0.33 for 51-60 years 0.66 decade to decade 61-70 years, 0.37 for 71-80 years group, 1 for patients over 81 years, and over the whole group studied, O: E ratio is 0.55.

**Fig. 2:**
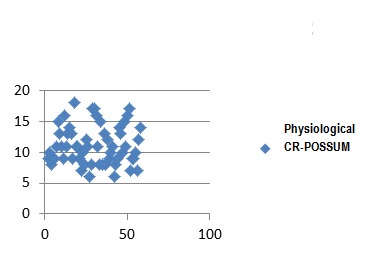
Physiological CR-POSSUM

**Fig. 3:**
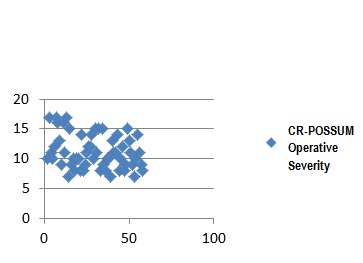
CR-POSSUM Operative Severity

**Fig. 4:**
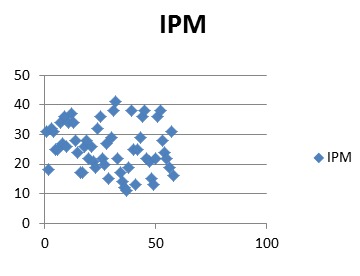
IPM

The number of patients who died by the 28th day after the operation was 10. Expected mortality by CR-POSSUM score was 31.03%, while the mortality rate was calculated to be 17.24%.

 Using statistical software “sofastatistics open for all", applying ∘χ test, it can be argued that there is a statistical significance between the CR-POSSUM physiological score and Mannheim score (p = 0.0035, p <0.01) for the study group.

 Applying χ2 test for forecasting mortality and deaths, the resulting statistical correlation (p = 0.026, p <0.05), with a specificity of 0.55 and a sensitivity of 0.69 for CR-POSSUM score. In Table 4 there are the results of the unpaired t test to compare variables age, organ failure, malignancy, generalized peritonitis, exudates faeces and duration of hospitalization, surgery deaths and possible correlation between them.

**Table 4:**
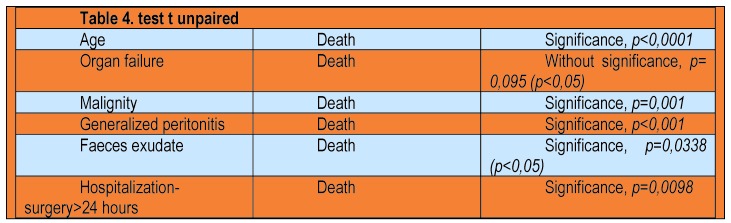
Simple linear regression of functional and activity indices

Thus it can be argued that there is a correlation between the patient’s age and death (p <0.0001), as can be said of the presence of malignancy compared to the number of deaths (p = 0.001) or the presence of exudate faeces and deaths (p = 0.0338, p <0.05). There is a correlation in terms of statistical supported from organ failure and the number of deaths (p = 0.095, p <0.05).

 Scoring Mannheim and the placement of each subgroup in the study group led to the following results: 8 (13.8%) patients who had healed Mannheim score ≥ 24, 40 (68.96%) patients healed with score> 24, 2 (3.44%) deaths for a score ≥ 24 and 8 deaths (13.79%) with a score> 24. All deaths occurred in patients older than 51 years. Regarding time interval until operator, there were six deaths in patients for whom surgery took place within 24 hours and 4 deaths in patients who were operated after more than 24 hours of hospitalization.

 Analyzing mortality in groups of patients according to age, hospitalization, surgery and IPM range, it has the highest value for patients over 61 years (22.5%), operated under 24 (22.2%) and IPM ≥ 24. The risk of death is higher in patients older than 61 years. What was noted in the study group was that the increased length of the interval admission-surgery has a high risk of death; the number of confirmed deaths is higher for the group of patients who had an intervention in the first 24 hours. This could be explained if we consider the overall condition of these patients on admission and associated ills.

 Mannheim average score was significantly higher in the group of patients who died (31.8 ± 5.37) compared with patients healed (21.24 ± 7.92, p = 0.00016). To score CR-POSSUM operative severity, the average was also significantly higher in patients who died (13.5 ± 3.2) compared with the patients healed group (10.87 ± 2.61, p = 0, 0085).

**Table 5:**
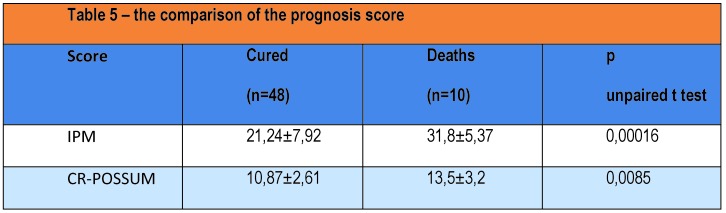
Comparison between functional and disease activity subgroups

## Conclusions

Based on these data, we can estimate that in patients with abdominal sepsis colonic origin, there is an overestimation of mortality by the application of CR-POSSUM score.

 Emergency surgery, the degree of peritoneal contamination and age are key variables to calibrate the CR-POSSUM score. There is a statistical correlation between age, malignancy, interval admission-surgery, generalized peritonitis and death rates. The statistical analysis was performed highlighted the prognostic value strictly related to the existence of a correlation between CR-POSSUM scores and score Mannheim.

 The results of this analysis are somewhat different from some previously published studies [**2-6**]. Over-prediction of mortality can be explained in part by the characteristics of mathematical scoring system (e.g. young patients undergoing elective interventions). Another possible explanation could be the existence of differences between subjects. There are patients who have a high degree of disease, and this has important implications in the development of the score.

 Patients with advanced colon cancer had an important nutritional deficiency. Malnutrition due to decreased nutritional intake, malfunctioning metabolic processes, hormonal abnormalities and related cytokines are the main causes of severe sepsis in cancer patients added [**7**]. In addition, it is possible that CR-POSSUM is overstated in elderly patients with preexisting comorbidities. The study group meets thus the situation for patients aged between 71 and 80 years. This situation is similar to that described by Slim et al. [**8**] who studied risk prediction score POSSUM and index AFC (Association Française Surgery).

 In a study comparing the POSSUM and CR-POSSUM scores, Horz M. et al [9] revealed over-POSSUM, compared with CR-POSSUM score which was more accurate, but the study was performed in patients undergoing colorectal surgery provost in general.

 It can be appreciated that the introduction of additional parameters of cardiac and pulmonary risk might improve the prognostic value for CR-POSSUM score. The association has verified the scores achieved, the aim is to increase the accuracy of forecasts.

 However, marking systems tend to optimize the data obtained in the study group. Although the CR-POSSUM score using data both to calculate and to validate the results, it is still important to check its validation and application in various populations.

 The results of this study demonstrated that both CR-POSSUM score and Mannheim have prognostic value for patients with abdominal sepsis in colonic peritonitis, both excluding scores surgery. Although both scores are based on clearly defined variables available, there are also differences. The association of several system scores in the case of CR-POSSUM and score Mannheim, do not only increase the prognostic accuracy of the evolution of patients with abdominal sepsis and provide a meaningful vision, for both the patient and the surgeon on surgical risk, while allowing a comparison of the types of surgery and medical care.
